# Monitoring Redox Processes in Lithium-Ion Batteries by Laboratory-Scale Operando X-ray Emission Spectroscopy

**DOI:** 10.1021/acsami.3c18424

**Published:** 2024-03-19

**Authors:** Abiram Krishnan, Dong-Chan Lee, Ian Slagle, Sumaiyatul Ahsan, Samantha Mitra, Ethan Read, Faisal M. Alamgir

**Affiliations:** School of Materials Science and Engineering, Georgia Institute of Technology, Atlanta, Georgia 30332, United States

**Keywords:** X-ray absorption, X-ray emission, spin state, lithium-ion battery, operando

## Abstract

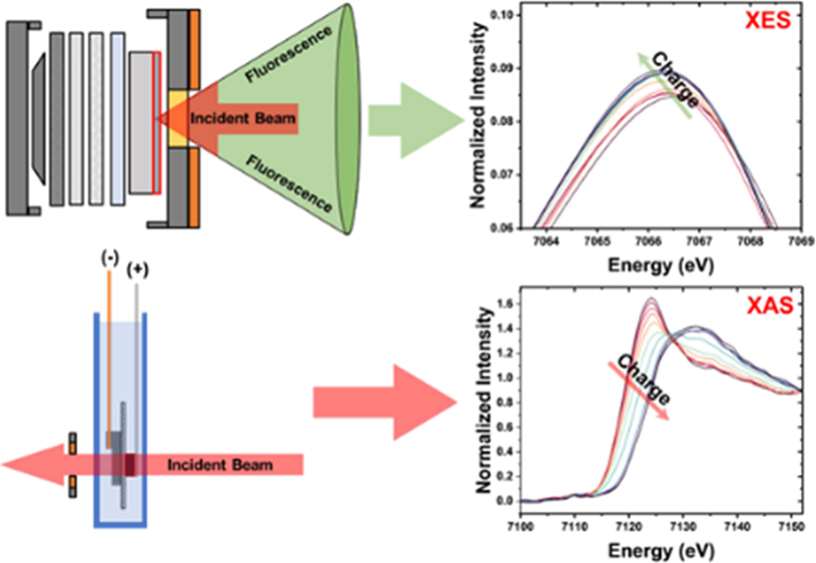

Tracking changes in the chemical state of transition metals in alkali-ion batteries is crucial to understanding the redox chemistry during operation. X-ray absorption spectroscopy (XAS) is often used to follow the chemistry through observed changes in the chemical state and local atomic structure as a function of the state-of-charge (SoC) in batteries. In this study, we utilize an operando X-ray emission spectroscopy (XES) method to observe changes in the chemical state of active elements in batteries during operation. Operando XES and XAS were compared by using a laboratory-scale setup for four different battery systems: LiCoO_2_ (LCO), Li[Ni_1/3_Co_1/3_Mn_1/3_]O_2_ (NMC111), Li[Ni_0.8_Co_0.1_Mn_0.1_]O_2_ (NMC811), and LiFePO_4_ (LFP) under a constant current charging the battery in 10 h (C/10 charge rate). We show that XES, despite narrower chemical shifts in comparison to XAS, allows us to fingerprint the battery SOC in real time. We further demonstrate that XES can be used to track the change in net spin of the probed atoms by analyzing changes in the emission peak shape. As a test case, the connection between net spin and the local chemical and structural environment was investigated by using XES and XAS in the case of electrochemically delithiated LCO in the range of 2–10% lithium removal.

## Introduction

Lithium-ion batteries (LIBs) gained remarkable popularity as a solution for storing and releasing energy reversibly for applications ranging from portable electronics to electric vehicles.^1^ The implementation of novel electrode materials in battery systems is aided by an understanding of the redox processes under operating conditions.^2^ Optical and infrared methods have been used to study battery systems^3^ but suffer from the high absorbance of photons in and photons out by the battery components. Methods that utilize X-rays, on the other hand, are highly desirable for the potential for high transmission into and out of working batteries, thereby enabling the interrogation of electrode material during battery operation.^4^ Furthermore, X-ray methods based on the excitation of bound electrons (core-hole spectroscopy) are element-specific and are, therefore, critical to keeping track of changes in the local chemical and structural environment around the constituent elements during the operation of battery systems.

**Figure 1:**
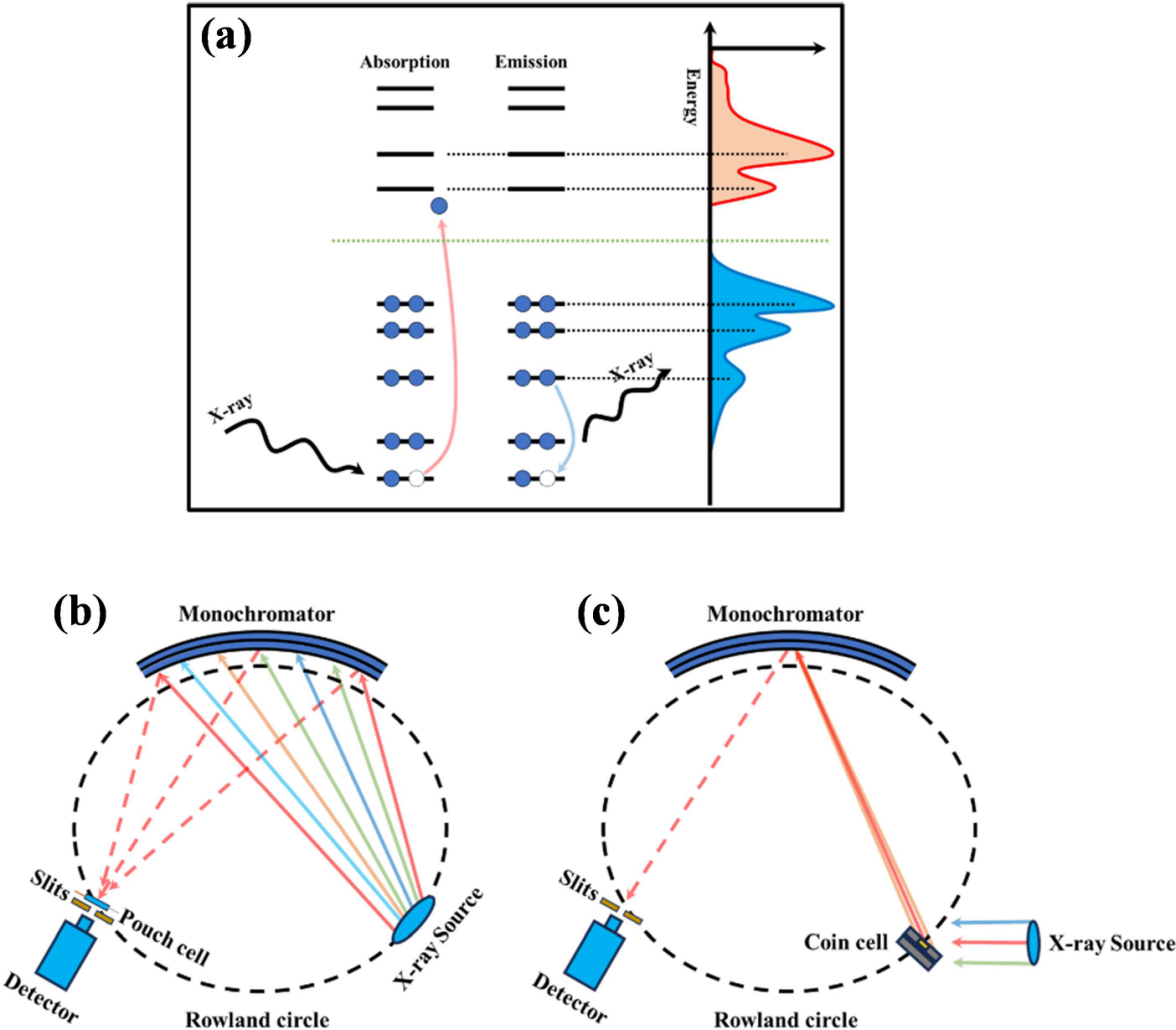
(a) Energy scheme depicting absorption and emission events. Absorption measures the process of generation of core holes, while emission measures the process of quenching generated core holes. Instrumental setup for (b) XAS and (c) XES measurements using a lab-scale instrument based on Rowland geometry (schematic adapted with permission from refs (12 and 37)). Copyright 2014 AIP publishing. Copyright 2021 American Chemical Society.

X-ray absorption spectroscopy (XAS) is one such core-hole method that measures the X-ray absorption coefficient of a sample as a function of photon energy through the excitation of inner-shell electrons (Figure 1a). Element specificity, chemical state sensitivity from the XAS near-edge structure (XANES), and sensitivity toward the local atomic structure through the XAS extended X-ray absorption fine structure (EXAFS) allow us to use XAS experiments to track changes in transition metals during the charge/discharge process in a battery in the absence of competing effects. Operando (real-time) XAS experiments for batteries help us understand the role of individual elements in the redox processes during cycling through oxidation state information. Past studies have extensively used XAS to investigate the redox mechanisms in transition metal oxide-based battery systems^5−11^ under both ex situ and operando conditions. X-ray emission spectroscopy (XES), a complementary technique to XAS is obtained by capturing the characteristic X-ray photons resulting from quenching generated core holes (Figure 1a). Element-specific emissions of sufficient energy resolution are simple to process and are sensitive to the chemical state, local structure, and spin state of the transition metal, making them an alternate method to XAS for studying the redox processes in batteries.

As opposed to ex situ approaches, operando characterization captures kinetically limited phenomena closer to real-time battery operation. The high flux requirement of such experiments often limits these studies to synchrotron sources. The limited access to synchrotron facilities has led to the development of laboratory-scale XAS and XES instruments using flux from bremsstrahlung sources and energy discrimination from single crystal diffraction, allowing us to explore redox processes in a battery. These spectrometers in these instruments are either based on scanning setups which employ the Rowland circle geometry^12−14^ or dispersive type which employs the von Hamos geometry.^15,16^Figure 1b,c shows the lab-scale setup based on the Rowland circle geometry used in this study to measure the absorption and emission spectra for battery systems. Laboratory-scale XAS/XES instrument can be further improved through the development of brighter sources and detectors with high resolving power enabling higher time resolution.

The Kβ_1,3_ and Kβ′ X-ray emission peaks in 3d transition metals involve 3p-to-1s transitions and obtain chemical sensitivity through interaction between 3p core electrons and electrons in the valence band.^17,18^ These exchange interactions make these emission lines sensitive to the valence shell’s electronic structure such as total spin and occupancy, the latter allowing chemical state speciation of transition metal-based compounds.^19,20^ Additionally, the spin sensitivity of the Kβ_1,3_ feature of XES allows us to explore changes in magnetic properties as a function of lithium removal in LIBs. Higher energy valence to core (VtC) emission lines are rich in information about the chemical state and ligand environment around the transition metal but have a low X-ray cross-section and require background removal from nearby, large peaks. These Kβ_2,5_ valence to core transitions involves the filling of metal 1s core holes from 3d valence levels and have been used in past studies to explore oxidation state, nearest neighbor distances, protonation, and hybridization.^21−24^ The abundant information obtained through the multiple XES emission lines can therefore be used, with minimal data processing, to understand redox chemistry in batteries as well as the connection between redox chemistry and magnetic properties.

The local chemistry changes in a battery electrode induced by charging/discharging, the resulting changes in the equilibrium local structure, and the connection of each of these effects on its magnetic properties, are expected to be complicated but scientifically interesting. The late John Goodenough, cowinner of the 2019 Nobel Prize in chemistry for the development of LIBs, noted the nonobvious connections between magnetic properties and the chemical environment half a century ago.^25^ Past studies on mixed transition metal oxide-based cathodes such as Li[Ni_1/3_Mn_1/3_Co_1/3_]O_2_ (NMC111) show that they exhibit an anomalous increase in magnetic moments after 50% lithium removal which could not be explained solely by the oxidation of nickel, manganese, or cobalt, suggesting that electron holes were being formed at oxygen sites.^6,26^ A sister compound to the NMC, LiNbO_2_ shows the formation of electron holes at oxygen sites with Li removal and reinsertion that can reversibly change its resistivity and give rise to potential memristive devices.^27^ LiCoO_2_ (LCO) cathodes, also a structural sibling of NMCs, show large resistivity changes with delithiation.^28^ Given their compositional and structural similarities to NMCs and based on past studies suggesting the localization of electron holes in oxygen sites during its deilithiation,^29^ LCO is expected to have anomalous magnetic behavior as well. Imanishi et al. through their work on XRD and NMR of chemically delithiated LCO revealed the presence of a new conductive hexagonal phase at 8% lithium removal.^30^ We expect such a phase to be indicated by an alteration in the electronic/spin structure (magnetic properties) local to cobalt and oxygen. Past studies have also explored LCO through magnetometry but have limited information on the first 10% of lithium removal.^31−34^ Since XAS and XES can be used in a complementary way to probe changes in the chemical state, interatomic distances, and spin states, these techniques can be used to explore LCO in the first 10% lithium removal to understand these dynamic changes.

In this study, spectral shape changes of Kβ_1,3_ and Kβ′ features of XES are utilized to track chemical state changes of transition metals in real-time and study redox processes using lithium half-cells comprising the following cathode materials: LiCoO_2_ (LCO), Li[Ni_1/3_Co_1/3_Mn_1/3_]O_2_ (NMC111), Li[Ni_0.8_Co_0.1_Mn_0.1_]O_2_ (NMC811), and LiFePO_4_ (LFP). We then present a side-by-side comparison of tracking lithium removal using Kβ_1,3_ XES versus the measurements by K-edge XAS, which serves as a benchmark. Further, VtC emissions of XES are utilized to study the change in chemical and ligand environments of cobalt with different amounts of lithium removal in LCO cathodes. Additionally, ex situ samples prepared using electrochemical lithium removal of LCO in the range of 2–10% are investigated using XAS and XES to understand the changes in the chemical state, interatomic distances, and spin state, resulting in the presence of a new conductive phase.

## Experimental Section

### Cell Assembly

**Figure 2:**
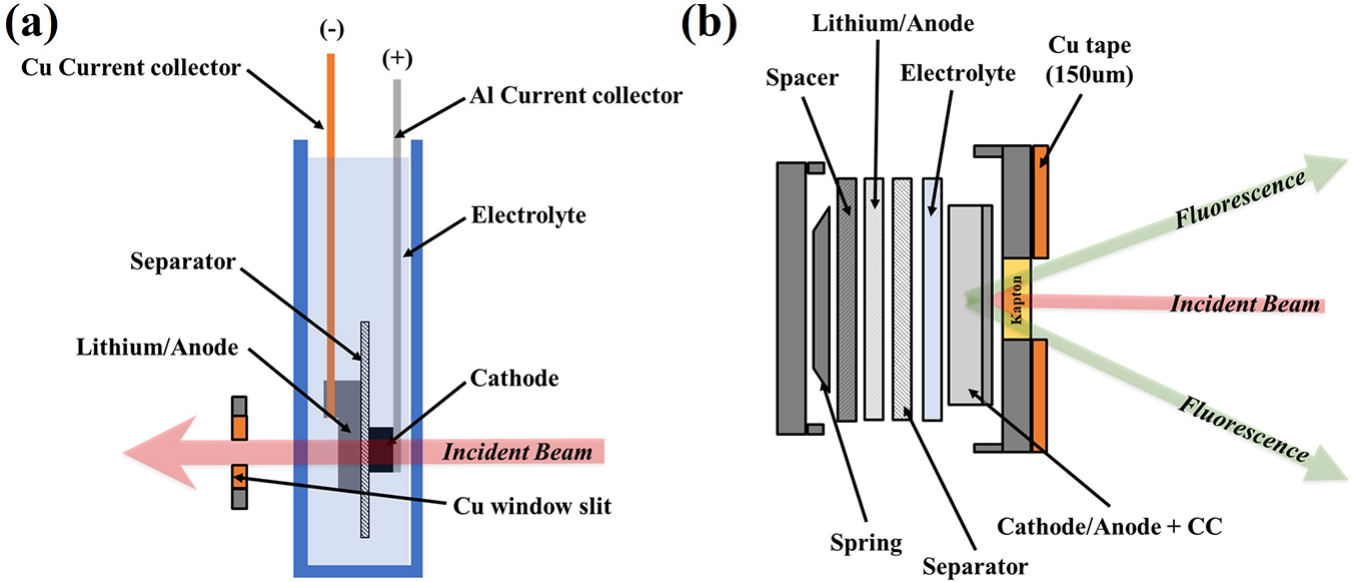
Cell design for operando measurements using (a) transmission mode XAS and (b) XES. Copper foil is used to block iron signals from cell components during XES measurements and define the transmission window for XAS measurements.

The cathodes were prepared based on a slurry-casting method. The active material was mixed with conductive carbon black and polyvinylidene difluoride (PVDF) with a mass ratio of 8:1:1 to obtain a slurry using *N*-Methyl-2-pyrrolidone as the solvent. The slurry was coated on aluminum foil using a doctor blade and dried overnight in a vacuum oven. The lithium half-cells were assembled inside an argon-filled glovebox using a CR2032 type coin cell (20 and 3.2 mm are the diameter and thickness of the coin cell) and pouch cell setup for XES and XAS as shown in Figure 2. A polypropylene membrane (Celgard 2500) was utilized as the separator along with 1 M LiPF_6_ in ethylene carbonate/diethyl carbonate with a volume ratio of 1:1 as the electrolyte for lithium-ion battery assembly. The half-cells were cycled under a C-rate of C/10 (charge/discharge the cell in 10 h.) during operando XAS/XES measurements using a potentiostat as shown in Figure S1. The corresponding voltage profiles for operando cycling are shown in Figure S2.

### XAS/XES Measurements

The Kβ fluorescence along with K-edge XAS (transmission mode) for Fe, Ni, and Co present in cathode samples was collected using a lab-scale instrument under operando/ex situ conditions. The operando spectra were collected with an interval of 1 h between scans for different cathode materials cycled at a C-rate of C/10. O K-edge near edge X-ray absorption fine structure (NEXAFS) for ex situ LCO samples were collected using beamline 7-ID-1 at NSLS-II at Brookhaven National Laboratory.

### Data Analysis

**Figure 3:**
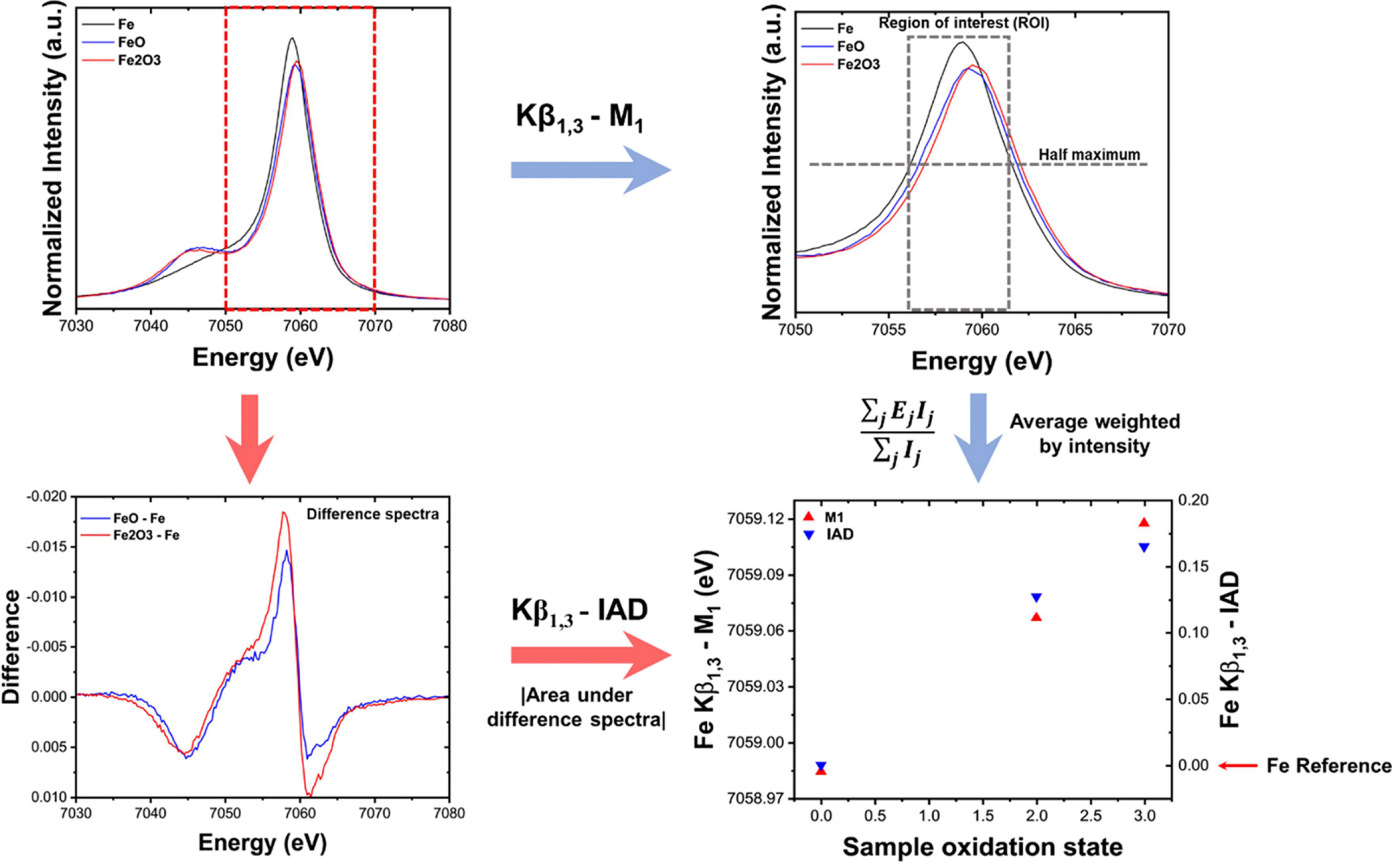
Schematic showing the calculation of Kβ_1,3_-*M*_1_ and the IAD from raw data for different iron oxides. Note that *M*_1_ calculations involve the region above half-maximum (ROI) while IAD involves the entire Kβ spectra. IAD and *M*_1_ of the area normalized Kβ_1,3_ feature is found to shift toward higher values with an increase in formal oxidation state of iron.

XAS data were normalized using Athena^36^ from the Demeter software package before the analysis of K-edge positions. The edge position was determined by using the maximum of the first derivative of normalized absorption. Bond distances were obtained from Fourier-transformed ex situ EXAFS (extended X-ray absorption fine structure). Kβ_1,3_ XES data were normalized based on the area under the peak. The integrated absolute difference (IAD) and first moments (*M*_1_) of the Kβ_1,3_ feature are summary statistics used to study changes in spin states.^20^ IAD is calculated as follows:
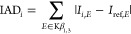
where *I*_*i*,*E*_ is the intensity of the spectra *I* at energy *E*. The first moment (*M*_1_) of the Kβ_1,3_ feature is estimated as follows:
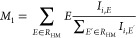
where RHM is the region where the intensity is above half of the maximum of the Kβ_1,3_ feature. Figure 3 shows the calculation of the IAD and *M*_1_ of the Kβ_1,3_ feature for different iron oxides from the area normalized spectra. We can use both the IAD and *M*_1_ of the Kβ_1,3_ feature to follow the chemical state of transition metals.

### Exchange Interactions in Kβ XES

To show the sensitivities of Kβ XES, we use the exchange energy difference as an example. The exchange energy difference between the Kβ′ and Kβ_1,3_^20^ which is dependent on the spin of the valence d shell is given by
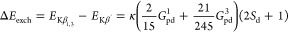
where κ is the scaling factor, *G*_pd_^1^ and *G*_pd_^3^ are the Slater–Condon parameters, and *S*_d_ is the spin of the d subshell. Kβ XES is sensitive to spin directly shown in this equation and oxidation state through the Slater–Condon parameters.^35^

## Results and Discussion

### Chemical State Speciation Using K-Edge XAS and Kβ_1,3_ XES To Study Battery Cathodes

**Figure 4:**
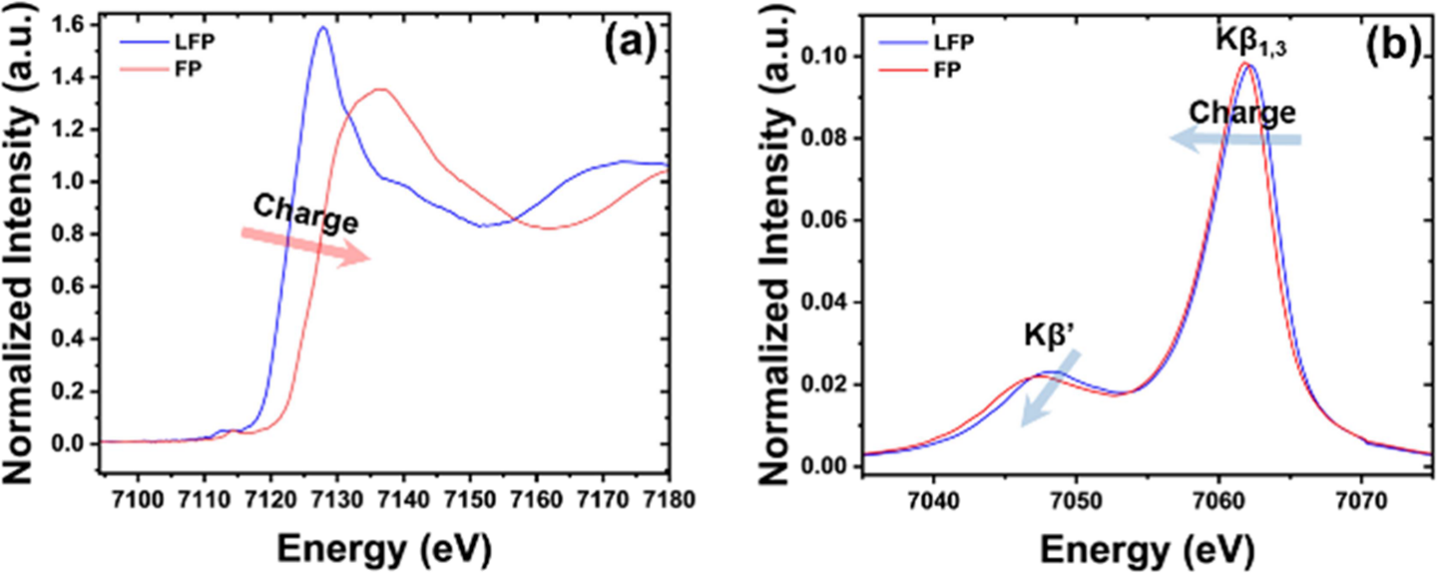
Changes in (a) Fe K-edge XANES and (b) Kβ_1,3_ XES between the charge and discharge state of LFP. Energy shifts between end states are greater for K-edge XAS compared to Kβ_1,3_ XES.

K-edge XAS for a 3d transition metal results from the excitation of the 1s core electron to unoccupied states. Information obtained from K-edge X-ray absorption near edge structure (XANES) helps us determine the oxidation state change of elements present in cathode materials during cycling in the absence of competing effects. For example, Figure 4a shows the shift in Fe K-edge toward higher energy along with a decrease in white line intensity during the charging of lithium iron phosphate (LFP) cathode indicating an increase in the average oxidation state of iron.

The event of electrons from the 3p shell filling core holes generated by X-ray absorption results in Kβ_1,3_ emissions which are sensitive to spin density around the metal atom through 3p-3d exchange-interactions^17,18^ that, consequently, also makes them sensitive to the chemical state of transition metal.^20^Figure 4b shows an example of shift in the Kβ_1,3_ mainline toward lower energy along with the decreased intensity of the Kβ′ region (∼7047 eV), indicating a change in the valence configuration of iron to reflect decreased total spin^20^ (oxidation of Fe) during the charging of LFP cathode materials. Smaller shifts between battery end states for Kβ_1,3_ emissions compared to K-edge XAS in 3d transition metals are primarily attributed to two factors. First, it is due to the lower sensitivity of the Kβ emission event (3p-to-1s) in 3d transition metals compared to K absorption (1s-to-4p) to change in screening of nuclear charge resulting from a change in valence electron population. Second, the exchange interactions which are responsible for most of the chemical state sensitivity of Kβ emissions are weaker than screening effects.^17^

Collecting XES along with XAS for the electrode material for LIBs as a function of lithium removal gives a better understanding of oxidation and spin state changes during energy storage and release by observing changes in occupied and unoccupied states. Trends in the spin structure of transition metal during electrochemical processes can be tracked using the IAD and *M*_1_ of Kβ_1,3_ emissions to monitor the redox processes and study changes in the magnetic properties of transition metal-oxide-based cathode materials during cycling.

### Noninvasive Electrochemical Monitoring Using Operando XES and XAS

**Figure 5:**
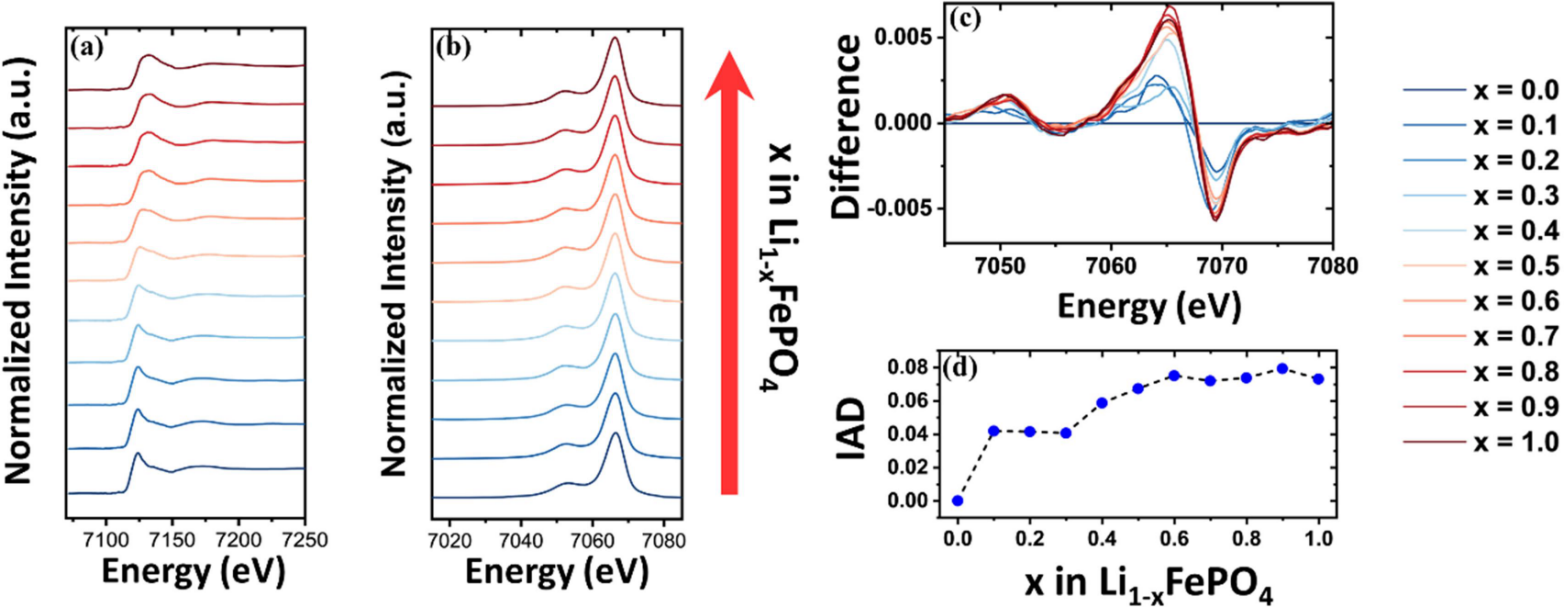
Operando (a) K-edge XAS and (b) Kβ_1,3_ XES for LiFePO_4_ half-cells along with (c) difference spectra and (d) IAD of the Kβ_1,3_ feature obtained under C/10 charging. Change in K-edge positions/IAD suggests a change in the oxidation/spin state of the transition metal.

With lithium metal as the counter electrodes, half-cells containing transition metal-based cathode materials such as LCO, NMC111, NMC811, and LFP were charged under a constant C/10 current. During the charge process, K-edge and Kβ_1,3_ XES were collected at intervals of 1 h. between scans for each transition metal species in the cathode as shown in Figure 5 for LiFePO_4_. The difference spectra along with the IAD values for Kβ_1,3_ XES were calculated by using pristine material (cathode material before charging) as the reference. IAD values for the Kβ_1,3_ feature of XES are found to increase with lithium removal in LiFePO_4_ marking oxidation of iron during charging. Spectral feature changes of XAS K-edge and XES Kβ_1,3_ (including difference spectra and the IAD) of other cathode materials (LCO, NMC111, and NMC811) are provided in Figures S4–S6 as part of Supporting Information.

**Figure 6:**
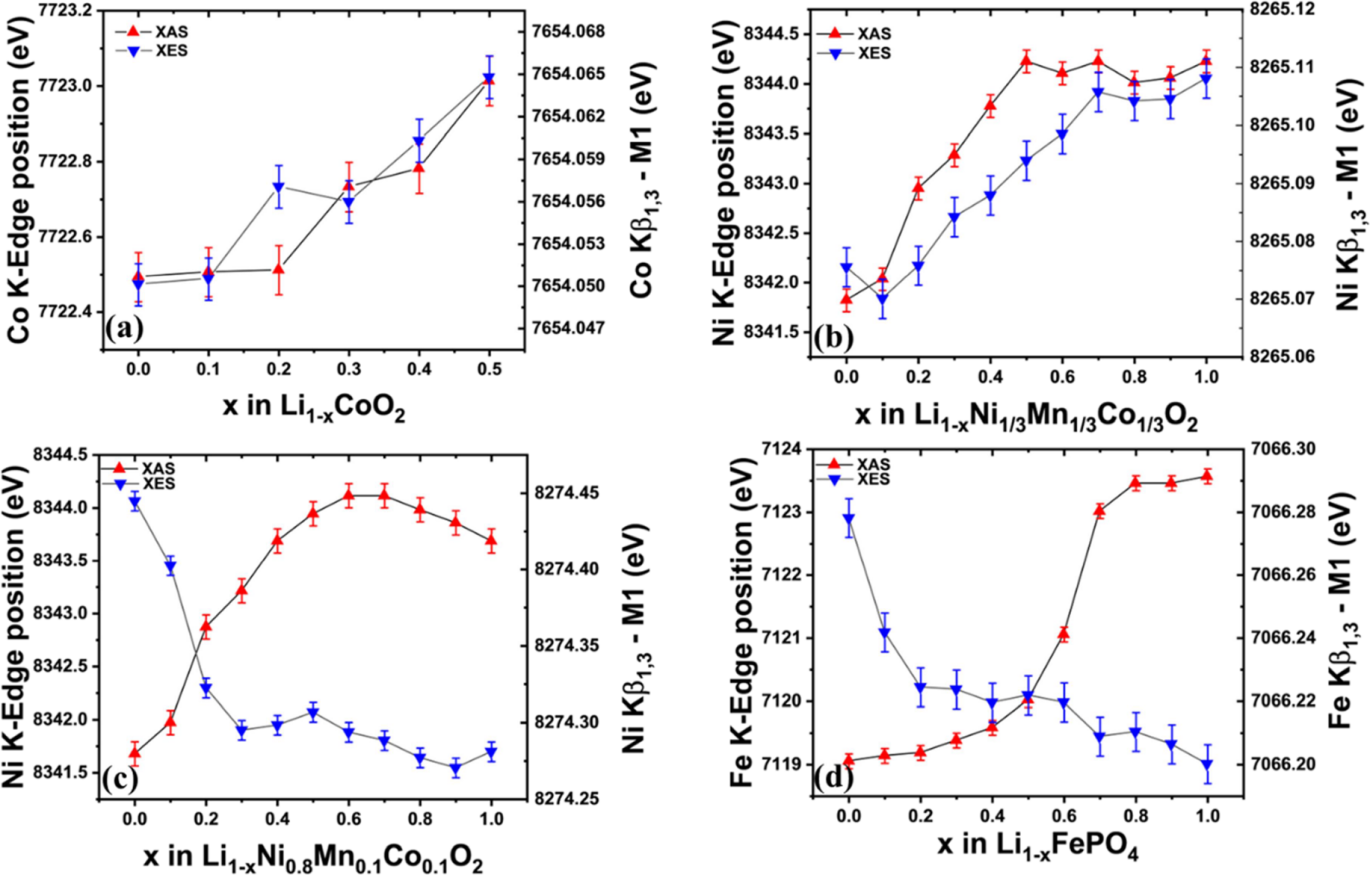
Comparison of chemical shift behavior between XES and XAS of transition metals during operando charging of (a) LCO, (b) NMC111, (c) NMC811, and (d) LFP. Kβ_1,3_-*M*_1_ feature shifts toward higher energies with lithium removal in the case of NMC111 but shifts toward lower energies in the case of NMC811.

The first moments of Kβ_1,3_ emissions and K-edge positions are calculated for spectra shown in Figures 5 and S4–S6 to compare XES against XAS for varying-cathode cells as a function of lithium removal (Figure 6). Error in the K-edge position and Kβ_1,3_-*M*_1_ of transition metal is calculated through maximum deviation from the mean which was obtained through ten consecutive scans of pristine pouch/coin cell (XAS/XES) before starting the experiment. Error bars obtained for pristine scans are utilized throughout the measurements of *M*_1_ and the K-edge of the transition metal, as the counts obtained during the operando experiment remain approximately the same for all measurements. The transition metal K-edge is observed to shift toward higher energies with lithium removal (resulting in oxidation of other species), whereas the shift experienced by the Kβ_1,3_ feature of XES is observed to shift either toward higher or lower energies with lithium removal. For LCO and NMC111 the Kβ_1,3_ feature shifts toward higher energies with lithium removal (Figure 6a,b), whereas for NMC811 and LFP the shift is toward lower energies (Figure 6c,d). This is due to the dependence of the exchange interaction between the 3p and valence orbitals of the 3d transition metal on the spin of the 3d shell. Shift toward higher energies indicates an increase in total spin and vice versa in the absence of other effects.^20^ Theories about the spin, oxidation state, and local geometry of the transition metal can be compared to experimental Kβ spectra through simulation using Crispy,^40^ a scientific software that utilizes multiplet models implemented in Quanty.^41−43^

During the charging of LCO, we observe a shift in cobalt Kβ_1,3_-*M*_1_ and K-edge toward higher energies with lithium removal (Figure 6a) with low activities in the initial 10% of lithium removal. This could be explained by the generation of electron holes at oxygen sites in this region.^5^ The first moments of Ni Kβ_1,3_ are observed to shift toward higher energies for NMC111, whereas it shifts toward lower energies for NMC811 during the charging of these cathodes (i.e., oxidation of Ni), suggesting an opposite spin behavior with a change in electronic structures (Figure 6b,c). In the case of LFP, the initial regions of lithium removal exhibit low K-edge sensitivity but high Kβ_1,3_ sensitivity making it more suitable to use emission spectra to monitor SoC in these regions (Figure 6d).

**Figure 7:**
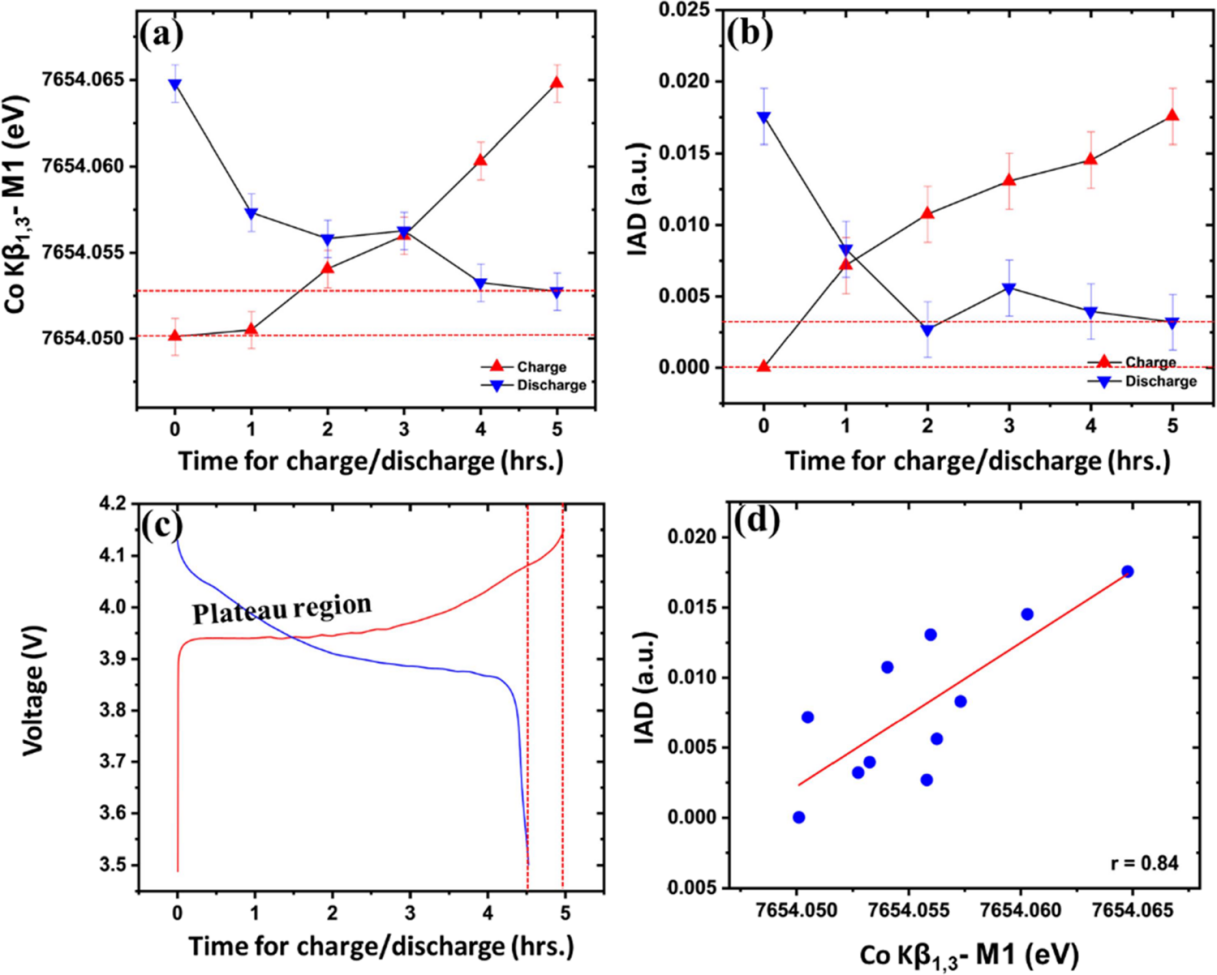
Operando monitoring of SoC using Co Kβ_1,3_ (a) *M*_1_ and (b) IAD for LCO half-cells cycled under a C/10 current along with (c) voltage profile obtained during charge/discharge. The (d) IAD plotted against *M*_1_ and their Pearson correlation coefficient (*r*). Red dashed lines indicate the difference between the charged and discharged state of LCO indicating the charge loss observed in the first cycle.

Figure 7a,b shows operando monitoring of SoC in LCO half-cells using *M*_1_ and IAD of Co Kβ_1,3_ spectra (summary statistics used to study the change in spin) during C/10 cycling (charge followed by discharge). Both measures of spin states of cobalt are distinct in the plateau region for LCO potentially allowing noninvasive tracking of SoC in these regions. The values of *M*_1_ and IAD do not return to their initial values after a full discharge making the difference between end states (red dashed lines in Figure 7a–c) a qualitative measure of irreversible charge losses during the first cycle of the electrochemical system, which is shown as relatively low Coulombic efficiency in the initial cycle(s) of battery electrodes (in our case the Coulombic efficiency is ∼90% for the initial cycle of LCO). The correlation between IAD and *M*_1_ for charge and discharge cycle in LCO is calculated through Pearson correlation coefficient (*r*) to be 0.84 (Figure 7d), indicating that IAD and *M*_1_ are fairly interchangeable, and using both for these data does not provide much additional information. Note that the correlation is calculated with a limited number of points in our case. Correlation plots for LFP, NMC111, and NMC811 are provided in the SI (Figure S7).

### Investigation of LCO Using VtC XES

**Figure 8:**
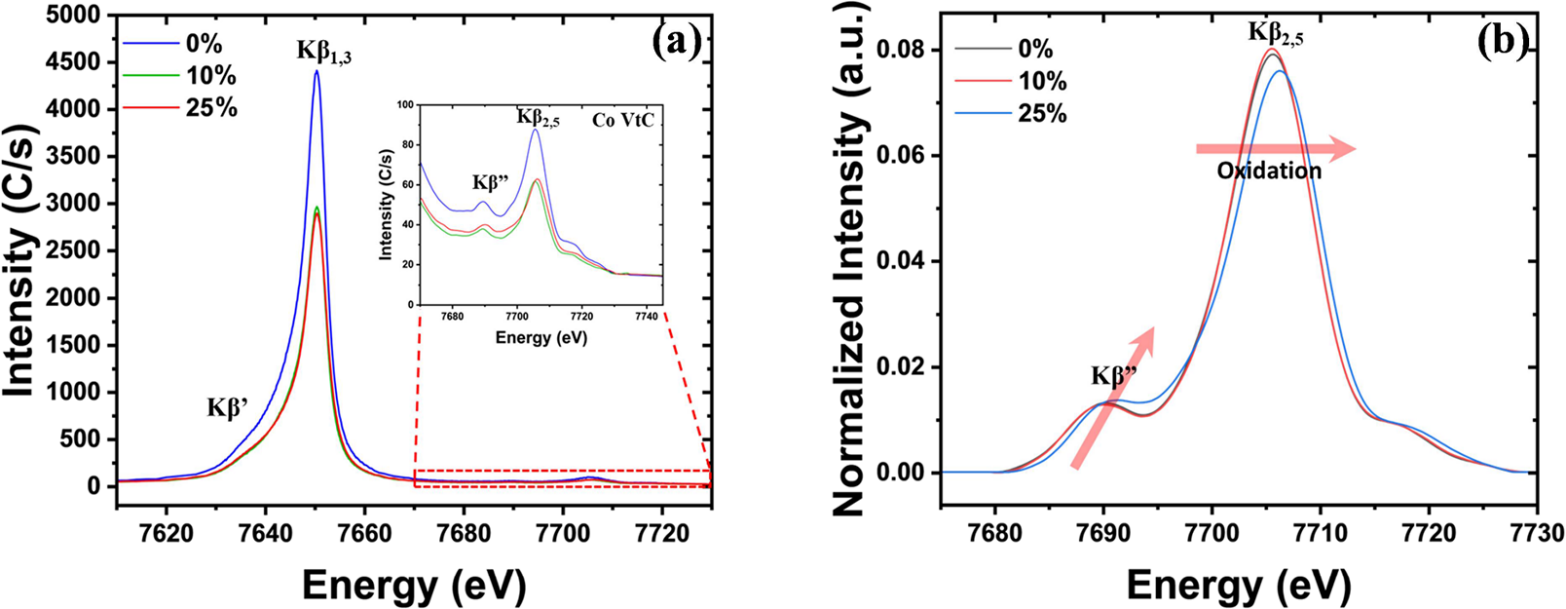
(a) Cobalt Kβ spectra including low cross-section VtC region expanded in the inset for LCO cathodes with different amounts of lithium removal. (b) Background-removed and area-normalized VtC spectra containing the Kβ_2,5_ and Kβ″ feature at 7705 and 7690 eV.

At a higher energy than the Co Kβ_1,3_ main line at ∼7650 eV are weaker transitions from the valence band to metal’s core orbitals (1s for K shell) denoted by Kβ_2,5_ and Kβ″ emissions. Figure 8a shows the Kβ spectrum, including the VtC region shown in the inset, for different amounts of lithium removal for LCO cathodes.

After removing the background using an end-points weighted method followed by area normalization^23^ (Figure 8b), we observe no shift in the peak position of Kβ_2,5_ feature between 0 and 10% lithium removal but an increase of 0.8 eV at 25% lithium removal suggesting an increase in the average oxidation state of cobalt. In addition, the intensity of the Kβ″ region is sensitive to nearest neighbor distances^17^ and is found to increase between 0 and 25% suggesting a decrease in Co–O interatomic distances. Note that the background of the Kβ_2,5_ spectra, arising from the Kβ_1,3_ mainline, must be removed, requiring these spectra to be treated prior to analysis. This step of background removal, if not performed properly, will lead to the introduction of systematic errors in the intensity and peak positions. Even with the low cross-section and the requirement of a background removal step for VtC emission, it is valuable to probe this region because it allows exploration of change in the chemical and local environment of transition metals with higher sensitivity compared to Kβ_1,3_ emissions.^18^

### Toward Decoupling Chemical State, Bond Distance, and Total Spin Information

**Figure 9:**
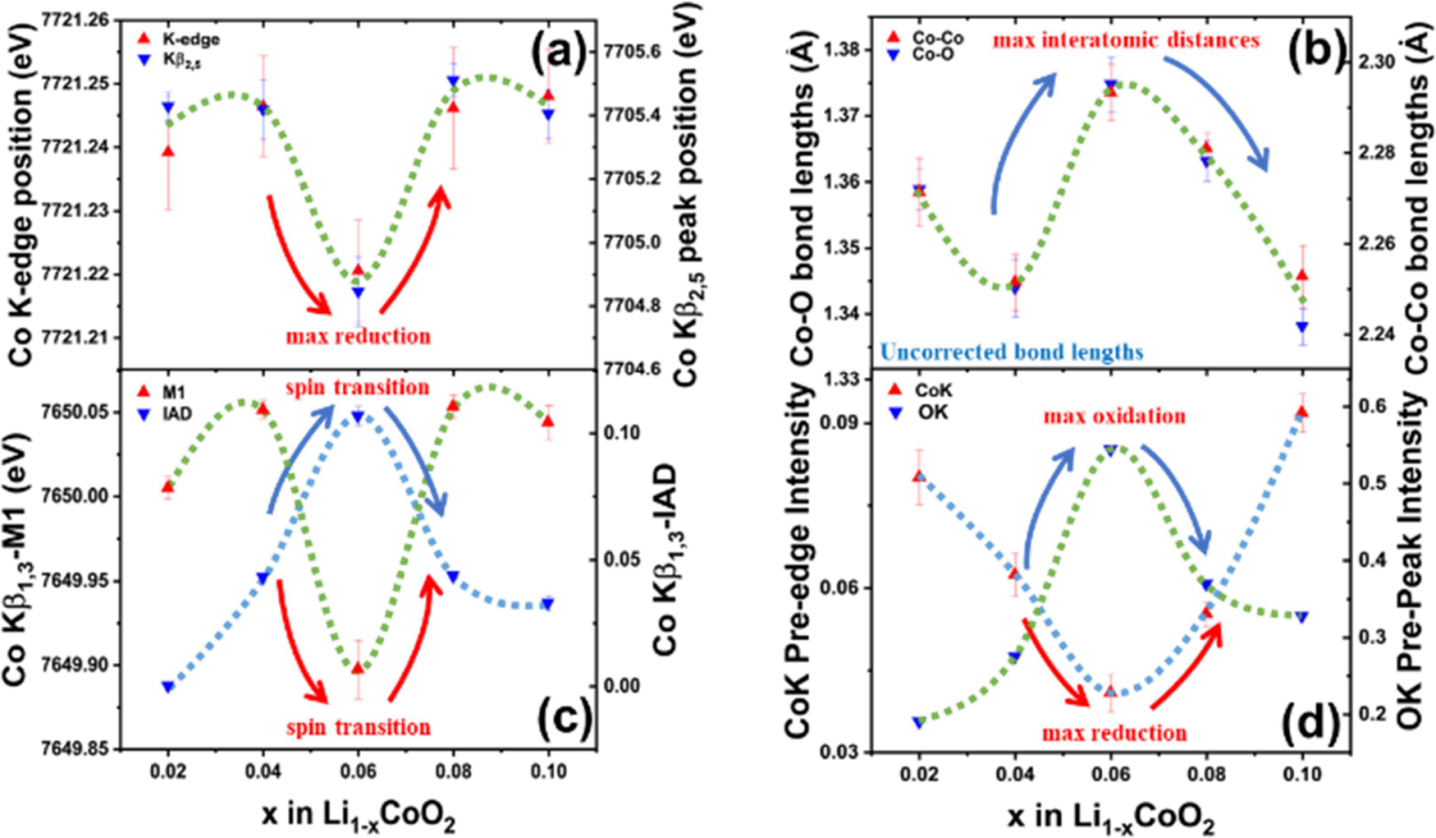
(a) Position of Co Kβ_2,5_ peak and K-edge indicates a decrease in the average oxidation state of cobalt at 6% lithium removal along with (b) increase in interatomic distances indicated through Fourier-transformed EXAFS. (c) Co Kβ_1,3_-*M*_1_ and the IAD (provides the absolute value of change in spectral features calculated using 2% removal as reference spectra) indicate a reduced spin state of cobalt at 6% lithium removal. (d) Intensity of the O K-edge’s prepeak feature (collected using a synchrotron source) and Co K-edge’s pre-edge feature suggests the generation of electron holes at the oxygen site responsible for anomalous spin activity in LCO. Note that the analysis results in this figure were made based on measurement data presented in Figure S3.

In order to explicate the particular sensitivity of XES to chemical state and net spin and how they are complementary to the chemical and local-structural information available from XAS, we consider the changes in the first 10% of Li removal in LiCoO_2_. Here, the chemical state, net spin, and atomic structure are causally interconnected and so the XES and XAS study should help us decouple the information. Ideally, LCO is diamagnetic (paired 3d-electrons) but is paramagnetic due to slight nonstoichiometry or a small amount of high spin Co^3+^ in equilibrium with low spin Co^3+^. Past studies using X-ray diffraction have indicated the presence of a secondary hexagonal phase arising at 8% lithium removal, which is distinct from the primary hexagonal phase present in pristine LCO.^30^ The presence and spread of this new phase will result from altering the electronic and local environments in LCO, both of which are observable through XAS and XES measurements. Ex situ samples prepared by electrochemical lithium removal of LCO cathodes in the range of 2–10% were selected to study these changes. Hard and soft XAS (and hard XES) signals of cobalt and oxygen, respectively, were collected as shown in Figure S3. Further analysis of the measurement results enabled us to suggest that LCO experiences a sharp change in electronic and local atomic structure at 6% lithium removal (Figure 9a–d). Cobalt K-edge XAS and Kβ_2,5_ XES (Figure 9a) indicate a decrease in average oxidation of cobalt at 6% allied with an increase in the Co–O and Co–Co interatomic distances (Figure 9b). Interatomic distances obtained through Fourier-transformed EXAFS are compared as measured (without model-based phase correction to avoid assumptions on the structure of LCO). Such observation is opposite to a typical trend (i.e., oxidation of cobalt with lithium removal) suggesting the presence of the new conductive hexagonal phase at 6% lithium removal.^30,39^ The localization of electron holes in oxygen sites at 6% lithium removal of LCO is shown through an increase in relative intensity of the prepeak feature of the O K-edge as shown in Figure 9d. Such phenomena for oxygen lead to an increase in the Pauling ionic size^38^ which agrees with the increase in lattice parameter along the *z*-direction for the new phase at 8% lithium removal.^30^ Reduction of cobalt at 6% (charge compensation through oxygen) results in reduced total spin detected through a shift in the Kβ_1,3_ feature of XES toward lower energy as shown in Figure 9c.

We have shown that with a relatively simple cell design, one can use XES, to follow the state of charge and the local magnetic properties of battery material during cycling. The most powerful use of XES is in combination with XAS to study any system where local chemistry can direct changes in the magnetic properties, as exemplified by our systematic study of Li extraction in LCO and the resulting spin transitions. The development of brighter sources along with detectors enabling us to have high resolving power (allowing us to discern features with ease) and low deadtime will allow access to regions of low cross-section (VtC emissions) in the time scale required for an operando experiment providing insights into the chemical state and local atomic structure changes during the cycling of batteries using laboratory-scale equipment.

## Conclusions

Operando XES is a powerful tool to study and monitor redox processes and magnetic property changes during the cycling of cathode materials for energy storage devices. In this study, we explored different battery systems using XES and benchmarked them against XAS. Additionally, XES and XAS are utilized to explore changes in the chemical, local, and spin states of cobalt resulting from the nucleation of a new phase in the case of LCO for 2–10% lithium removal. We predict that development in lab-scale spectrometers along with X-ray transmissive cell designs will allow reliable, accessible, and quick monitoring of both electronic and local atomic structure changes in energy storage devices through different emission lines.

## Supporting Information Available

Additional experimental details, voltage profiles, ex situ XAS and XES measurements of LCO in the range of 2–10% lithium removal, and operando XES and XAS for LCO, NMC111, and NMC811 half-cells (PDF)

## Abbreviations

XAS: X-ray absorption spectroscopy
XES: X-ray emission spectroscopy
XANES: X-ray absorption near edge structure
EXAFS: extended X-ray absorption fine structure
IAD: integrated absolute difference
*M*_1_: first moments
LIBs: lithium-ion batteries
VtC: valence to core emissions
