# Supplementary material for: Monitoring Redox Processes in Lithium-Ion Batteries by Laboratory-Scale Operando X-ray Emission Spectroscopy
Source: ACS Appl Mater Interfaces. 2024 Mar 19;16(13):16096–105. doi: 10.1021/acsami.3c18424 (PMC10995943; doi:10.1021/acsami.3c18424)
Supplement: Supplementary file 1 — am3c18424_si_001.pdf [file am3c18424_si_001.pdf]

Supporting Information for:

Monitoring Redox Processes in Lithium-ion Batteries using Laboratory-scale Operando X-ray Emission Spectroscopy

Abiram Krishnan, Dong-Chan Lee[†], Ian Slagle, Sumaiyatul Ahsan, Samantha Mitra, Ethan Read, & Faisal M Alamgir*

School of Materials Science and Engineering, Georgia Institute of Technology, Atlanta, Georgia 30332, USA.

Corresponding author

*faisal.alamgir@mse.gatech.edu

1. Instrumental setup for lab-scale XAFS/XES:

The lab-scale instrument (easyXAFS300+) allows us to perform both XAFS and high-energy resolution XES by changing the position of optical elements. In the XAFS mode of the spectrometer, polychromatic X-rays from the source are monochromatized by the spherically bent crystal analyzer (SBCA), and the resultant X-ray is transmitted through the sample to the detector (Fig. S1a). In the emission mode of the spectrometer, the source X-rays strike the sample first resulting in fluorescence from the sample which is monochromatized by the SBCA and passes through the detector (Fig. S1b).

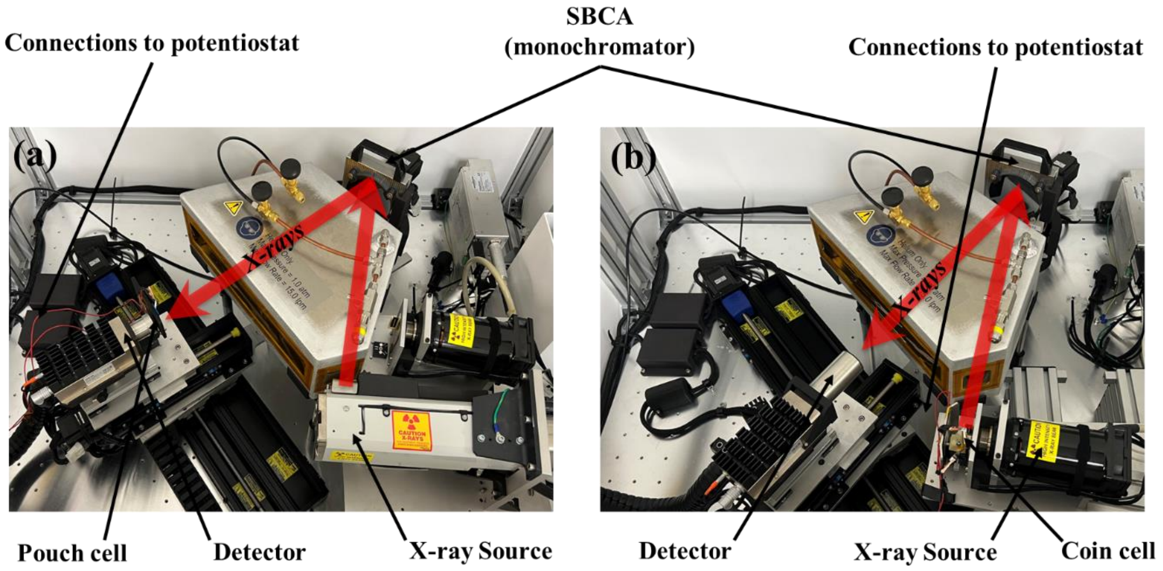

Fig. S1 (a) Interior instrumental setup for *operando* (a) XAFS and (b) XES measurement of pouch/coin cells.

2. Electrochemical data for operando cells:

A constant current charge/discharge experiment was employed to cycle batteries containing different active materials. A single-channel potentiostat by PINE instruments was used to charge the batteries during the collection of XAFS/XES spectra. The voltage profiles for different electrode materials under C/10 charging are shown in Fig. S2.

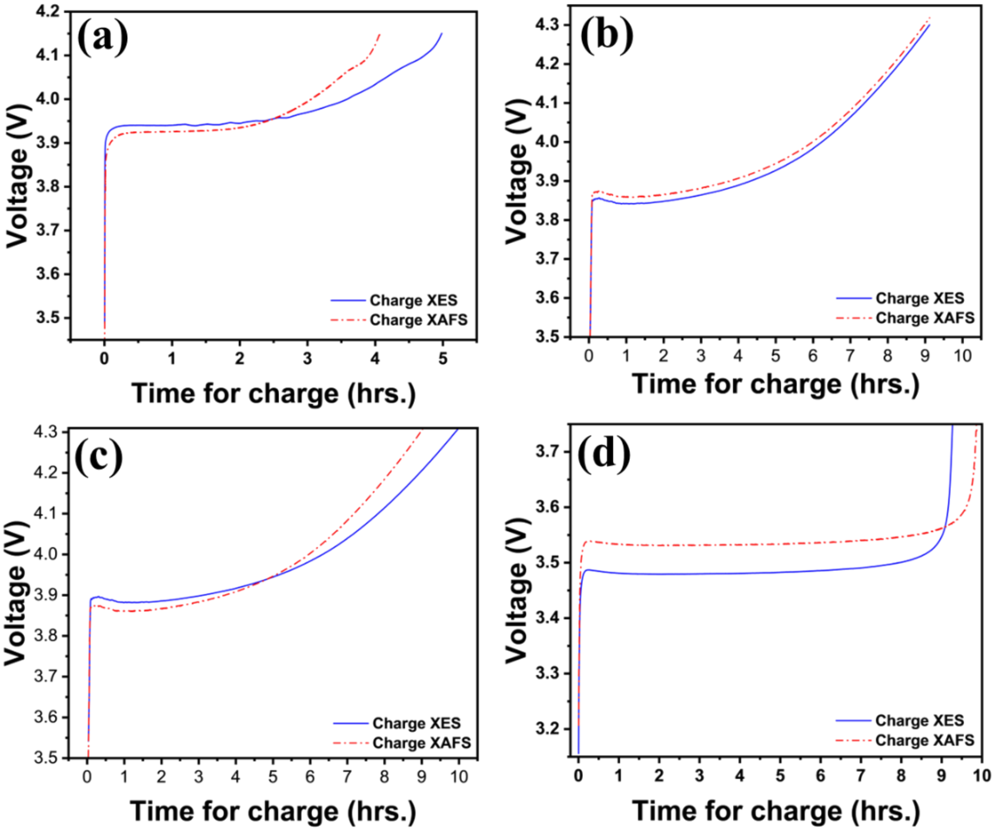

Fig. S2 Voltage profile for (a) LCO (b) NMC111 (c) NMC811 (d) and LFP during XES/XAFS measurements under a C/10 charge.

3. Ex-situ measurement of LCO cathodes using hard and soft X-ray techniques:

The change in spin/oxidation state, interatomic distances, and role of oxygen in LCO cathodes in the range of 2-10% delithiation is obtained through the measurement of cobalt Kβ emissions (including VtC), Co K-edge (including EXAFS) using lab-scale instrument followed by OK-edge measurements from beamline (Fig. S3). Background of VtC emissions resulting from the mainline were treated using an end point weighted background removal step in the energy range of 7670 - 7740 eV. Athena[36] from the Demeter software package was utilized to normalize cobalt K-edges before determining the edge positions through maximum of first derivatives. Uncorrected bond-distances were obtained from Fourier-transformed EXAFS region using a Hanning window. Table. S1 shows the various Athena parameters used to analyze cobalt K-edge for obtaining uncorrected interatomic distances.

Table S1. Athena parameters used in study to obtain uncorrected interatomic distances from cobalt K-edge.

**Pre-edge range**	7652.25 - 7703 eV
**Post-edge range**	7824.25 – 8004.25 eV
**Normalization order**	3 (quadratic)
**k – range**	2.6 – 7.8 A^{-1}
**k-weight**	2
**Spline range**	7724.25 – 8006.75 eV
**Rbkg**	1

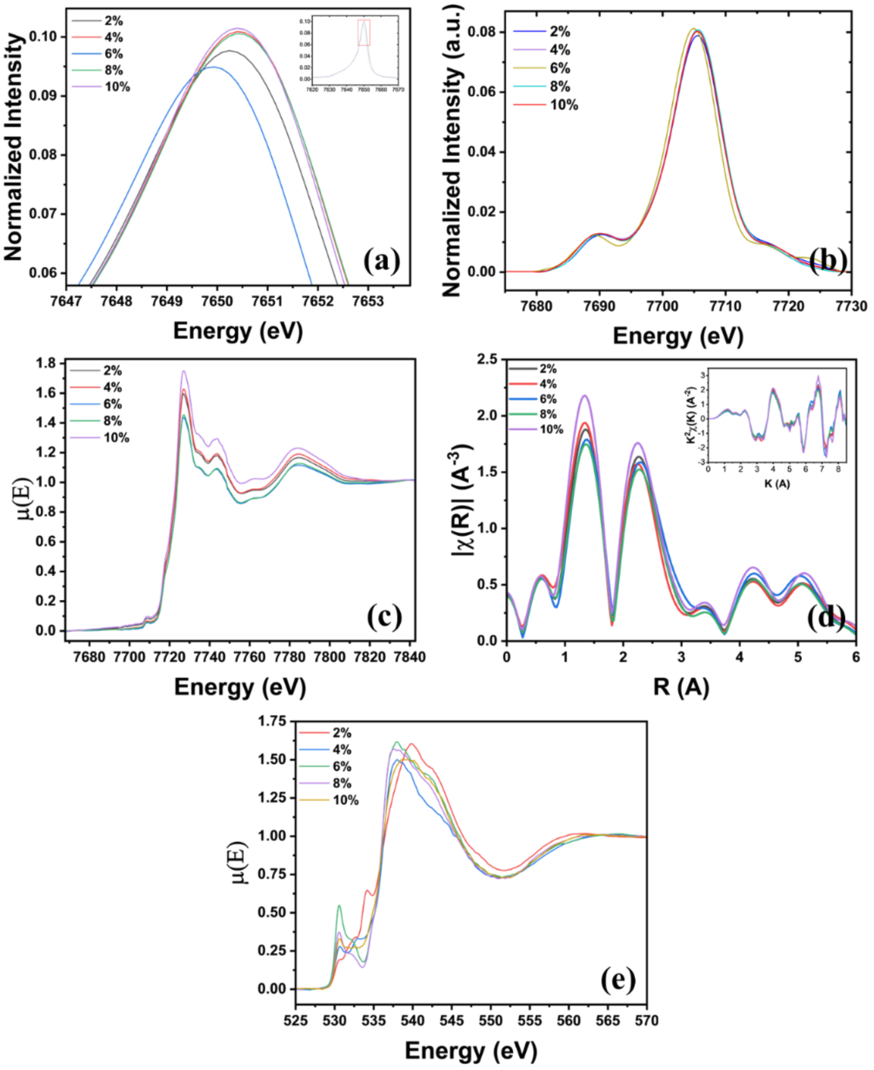

Fig. S3 ex-situ measurement of (a) Co Kβ$_{1,3}$ (b) Co VtC (c) CoK-edge (d) R-space EXAFS along with K-space provided in inset and (e) OK-edge for LCO samples (collected using synchrotron source) with 2-10% delithiation.

4. Operando XAS and XES measurements for LCO, NMC111, and NMC811

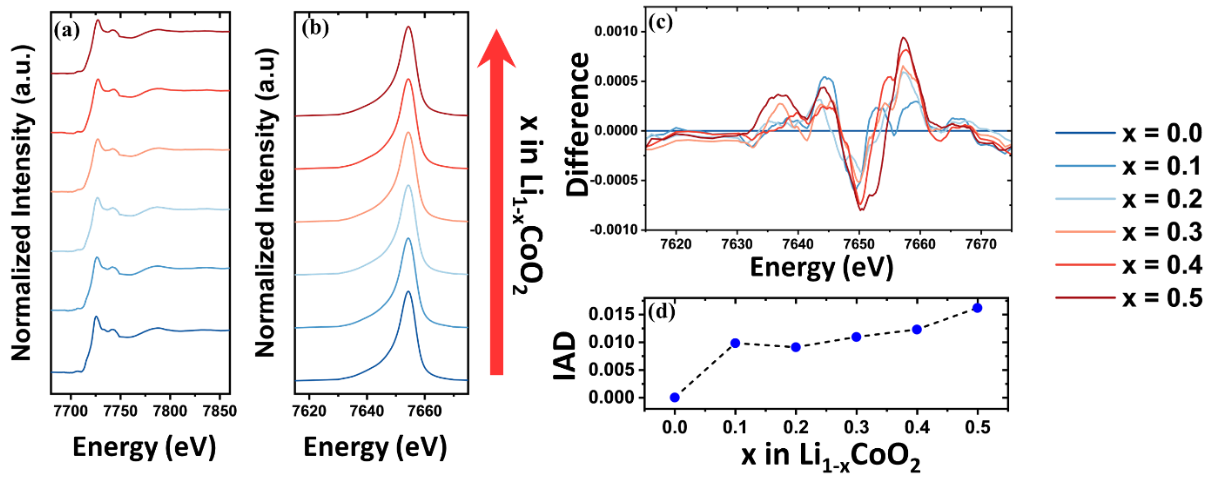

Fig. S4 Operando (a) K-edge XAS and (b) Kβ1,3 XES for $LiCoO_2$ half cells along with (c) difference spectra and (d) IAD of Kβ1,3 feature obtained under C/10 charging.

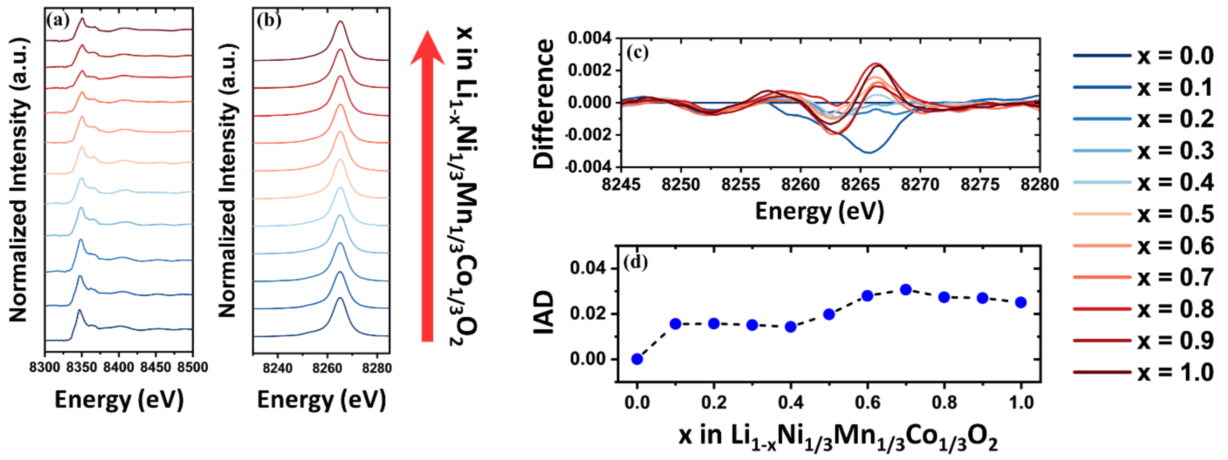

Fig. S5 Operando (a) K-edge XAS and (b) Kβ1,3 XES for NMC111 half cells along with (c) difference spectra and (d) IAD of Kβ1,3 feature obtained under C/10 charging.

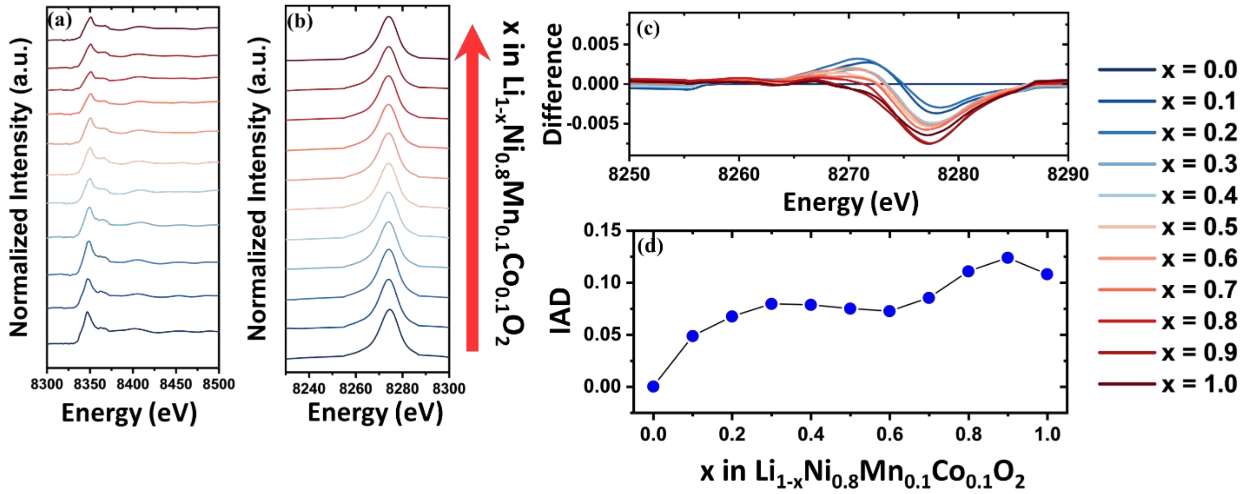

Fig. S6 Operando (a) K-edge XAS and (b) K$\beta_{1,3}$ XES for NMC811 half cells along with (c) difference spectra and (d) IAD of K$\beta_{1,3}$ feature obtained under C/10 charging.

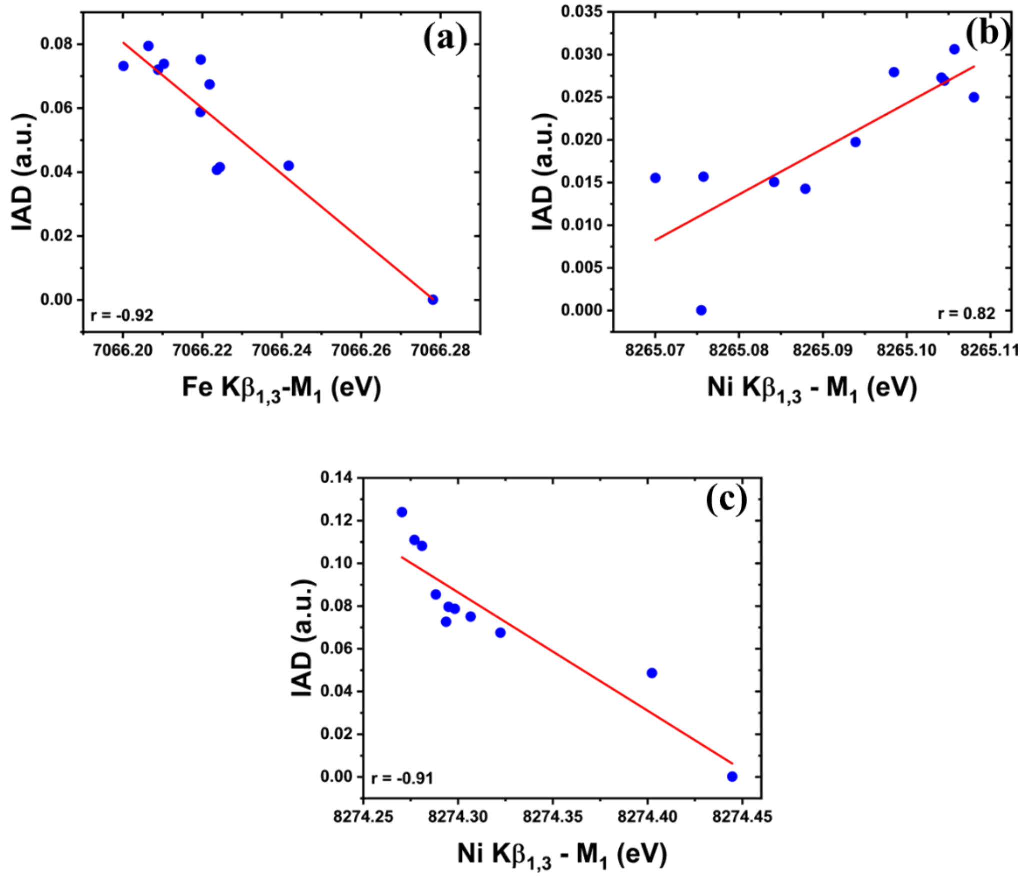

Fig. S7 IAD plotted against M_1 for (a) LFP, (b) NMC111, and (c) NMC811 to obtain Pearson's correlation coefficient (r) through linear fitting. The r value is not robust to outliers resulting in higher correlation for (a) and (c).
